# Influence of weather on the behaviour of reintroduced Przewalski’s horses in the Great Gobi B Strictly Protected Area (Mongolia): implications for conservation

**DOI:** 10.1186/s40850-022-00130-z

**Published:** 2022-06-09

**Authors:** Anna Bernátková, Ganbaatar Oyunsaikhan, Jaroslav Šimek, Martina Komárková, Miroslav Bobek, Francisco Ceacero

**Affiliations:** 1 Faculty of Tropical AgriSciences, Czech University of Life Sciences Prague, Prague, Czech Republic; 2 Great Gobi B Strictly Protected Area, Takhin Tal, Mongolia; 3 Zoo Praha, U Trojského zámku 120/3, Prague, Czech Republic

**Keywords:** Activity budget, Arid environments, *Equus ferus przewalskii*, Gobi Desert, Soft-release

## Abstract

**Background:**

Reintroduction is a common technique for re-establishing threatened species. However, the adaptation to novel habitats with distinct conditions poses a risk of failure. Weather conditions affect the behaviour of animals, and thus, their adaptation to new conditions and survival. Reintroduced Przewalski’s horses living in Mongolia’s continental arid climate with extreme temperature and precipitation variability, serve as an ideal model species for studying the behavioural response of selected groups to these harsh conditions.

**Methods:**

The research was conducted in The Great Gobi B Strictly Protected Area, Mongolia. In summer 2018, three groups were recorded (Azaa, Tsetsen and Mares18) involving 29 individuals. In Spring 2019, 4 groups were recorded (Azaa, Tsetsen, Hustai1 and Mares19) involving 34 individuals. In Autumn 2019, 4 groups were recorded (Azaa, Tsetsen, Hustai2 and Tanan) involving 35 individuals. Thirteen weather variables were recorded in 10-min intervals, together with the percentage representation of selected behavioural categories (feeding, locomotion, resting, and social). The effect of weather on behaviour was analysed through GLMM. Influence of the group-history factors (recently reintroduced, long-term reintroduced and wild-born) was also analysed.

**Results:**

Feeding significantly increased with cloudy and windy conditions and was more frequent in autumn than spring and summer. Locomotion was positively explained by temperature and cloudiness and was higher in summer than spring and autumn. Resting behaviour decreased with altitude and cloudiness, and the dispersion of the group was lower when resting. Increased social interactions were observed with higher temperatures and were more frequent in summer compared to spring and autumn. Differences were found in the display of the behaviours among the selected harems, showing interesting patterns when grouping them according to their origin and experience.

**Conclusions:**

Weather patterns seem to influence the behaviour of Przewalski’s horse. These results might assist in further management plans for the species, especially in the view of intensifying climate change and alteration of weather patterns. As previously suggested, after approximately 1 year, horses adapt to novel conditions and display the typical behavioural pattern of wild-born Przewalski’s horses.

**Supplementary Information:**

The online version contains supplementary material available at 10.1186/s40850-022-00130-z.

## Background

Reintroduction is an attempt to bring back species to areas of their historical range, and it is a common technique for in situ conservation. It is one of the most effective conservation techniques for re-establishing and supporting threatened species populations, sometimes the only way, however they have a relatively low success rate [1–3]. Access to large enough areas of suitable habitat (especially important for larger species) and the genetic makeup of the reintroduced population are the most critical determinants of a reintroductions long-term success [2–4]. As a result of scarcity of such large suitable areas, some animal species, such as northern bobwhite (*Colinus virginianus*), roe deer (*Capreolus capreolus*), or Pzewalski’s horse (*Equus ferus przewalskii*) have been reintroduced to the edge of their original habitat [5–10].

Another obstacle in reintroduction is the lack of knowledge on the ecology of the reintroduced species prior to its disappearance from the native area [9–11]. In general, most reintroductions pose a risk of failure as they require the movement of animals from a relatively secure environment (such as zoos and breeding facilities) to a harsh environment which might often be located on the edge of their original habitat [12]. After the translocation animals require time and space in order to be able to recover from the stress of transportation and to maintain and develop key cognitive processes such as habitat utilisation, anti-predatory behaviours, social behaviours and territory or home range establishment [13–15]. Therefore, the preferred method of release for many reintroduced species worldwide, including mammals, is soft-release. It allows animals to become accustomed to its new environment, while they are still being fed and/or protected from predators [16–21]. Time spent in the acclimatisation facility allows animals to develop affinity to novel habitat as well as with opportunities to adjust to local environmental conditions [19, 22, 23].

Przewalski’s horse was listed as Extinct in the Wild from 1996, with the last individual seen in 1969 in the Guntamga spring of the Great Gobi B Strictly Protected Area (GGBSPA). Thanks to the ongoing reintroduction efforts the species is listed as Endangered from 2011 [11]. The Przewalski’s horse is a flagship species which could be used for conservation of the whole habitat [24]. But reintroduction into its former habitat and further conservation is fraught with challenges and requires immense effort [25].

At the GGBSPA, which is the part of Great Gobi Biosphere Reserve, the first reintroduced horses were released from acclimatisation enclosures in spring of 1997 and currently, there is a mixture of recently reintroduced, long-term reintroduced and wild-born animals (wild-born individuals are in majority [26];). At present, there are 349 Przewalski’s horses, involving 24 harems, and 3–5 bachelor groups. Two hundred thirty-one of the horses are females and 148 are males. From 1992 to 2019, a total of 131 horses were transported from the zoos and reserves into the GGBSPA. Of these, 36 were males and were 95 females. Of the currently living horses, 315 were born in the Gobi, the remaining 34 horses were translocated to the area (personal communication with Dalaitseren Sukhbaatar, Takhi researcher, GGBSPA). Inland continental and arid climate of the GGBSPA is defined by extreme temperature and precipitation variability through the year with temperatures varying from − 40 to 40 degrees Celsius. Extreme drought of the warm-season and snow cover limiting animals from grazing in the cold season can occur in the same year. Dzud (extremely severe winter) induced wild-horse mortality rates of 21% in 2000/2001 and of 60% in 2009/2010 (livestock mortality was even higher, 67% in 2009/2010). This is a huge challenge for small and isolated animal populations such as the reintroduced population of Przewalski’s horse [27]. Therefore, horses living in these extreme conditions serve as an ideal model species for monitoring the relationship between reintroduced animals and their environment.

In reintroduced and endangered species habitat use is one of the key studied factors [28, 29]. In equids, forage abundance is the most important determinant for habitat use because of their digestive anatomy [30, 31] and they may spend more than half of their daily time budgets grazing to get enough nutrients [32]. However, inconsistent and contradictory information has been described by previous observations of the feeding behaviour and daily budget of horses (feral horses [24–34]; wild horses [25, 32, 35]:). In Hustai National Park, Mongolia, harems of Przewalski’s horses usually feed in the morning and evening, before and after the walk to the water source [25, 35]. In the spring, summer and autumn, groups typically migrate up to higher altitudes to rest in shade during the day. In the cold season, they tend to relax on sunny south-facing areas and spend more time at lower altitudes [35]. Under semi-natural conditions, average feeding rate of Przewalski’s horses in spring is much higher than at any other period of year, and the level of activity is generally high. In summer, the feeding levels are lowest compared to the rest of the year and activity shifts to the night. In autumn, the level of activity and feeding is usually high. In winter, the average daily activity is lower than at any other period of the year and the level of feeding is usually high (12 Przewalski’s mares [24]).

It is apparent, that feeding behaviour of Przewalski’s horses is influenced by the seasonal changes in their habitat. The GGBSPA is considered to be the edge of the original habitat of, Przewalski’s horses [5, 7, 8]. In such dry habitat, they need to stay close to water points to be able to drink at least two times per day [10] and their behaviour is affected by this need [36]. Ranging behaviour of the Przewalski’s horses in this area is also widely fluctuating, forcing them to sustain excessive heat load. Only after the sunset and during cooler climatic conditions, they are able to move to more favourable feeding areas [37]. At the GGBSPA, there is a mixture of recently reintroduced, long-term reintroduced and wild-born animals [26], and behaviour and habitat use of the recently reintroduced animals is possibly influenced by the need to adapt to novel conditions as identified by Scheibe et al. [38]. The study of Scheibe et al. [38] analysed activity and feeding behaviour of the herd of Przewalski horses over a 2.5-year period in a semi-reserve in Europe. The observation cycle involved an adaptation to nature-like conditions in the first year. But only after the first winter in the semi-reserve, horses showed a similar annual trend to that observed by other studies aimed at the annual budget of Przewalski’s horses [38].

For the successful management of this endangered species, knowledge on its ecology considering possible differences of animals of different origins is vital. This need is exacerbated by the constant population growth, demanding larger areas of suitable habitat. There is a lack of research on the influence of weather conditions on Przewalski’s horse behaviour and, in addition, most of the published data is from captive populations. It is critical to have more information about the effect of environmental conditions on daily budget and habitat use of the horses translocated to the novel habitat and to apply such knowledge to the management of the species.

In this paper, we aimed to present the most important patterns of behavioural response of the Przewalski’s horse to selected weather factors in the highly demanding environment of the GGBSPA. We hypothesized that the response would differ according to the group origin and experience, as by previous research it was described, that after release, Przewalski’s horses do not present the typical behavioural pattern of wild individuals. Our finding could servefor the selection of future reintroduction sites, as climate change-altered habitats may become a new norm and the ability of appropriate response of the species to the changing environmental conditions may present the biggest conservation challenge in the future.

## Materials and methods

### Study area

The GGBSPA (established in 1975) is a part of the Great Gobi Biosphere Reserve. Currently, it encompasses over 18,350 km^2^ of desert steppe and desert habitat [26]. This protected area located in SW Mongolia is a reintroduction site for the Przewalski’s horse and an important refuge for several other endangered species [39, 40]. Despite its protected area status, the GGBSPA is used by about 130 families with close to 70,000 heads of livestock mainly in winter and during spring and fall migration [41].

The climate of the GGBSPA is continental and very dry. The temperatures differ significantly both during the day and night and between the seasons. The altitude ranges from 1100 to 2900 m above mean sea level (mamsl) and the annual mean temperatures are below zero (°C) [26]. The GGBSPA is situated between the Altai Mountains and borders with China. The amount of precipitation is within the typical range of semi-desert climate (around 150 mm per year). Most of the precipitation falls in the summer rains. However, not only the drought and the large temperature gradient, but also the enormous annual differences are characteristic for the habitat. Plants have to cope with water stress and a high temperature gradient. The high degree of mobility of animals is essential in order to be able to find suitable habitats depending on the situation [26].

### Data collection

Authorisation for working with this endangered species in the protected area was granted by the director of the Strictly Protected Area.

**Fig. 1:**
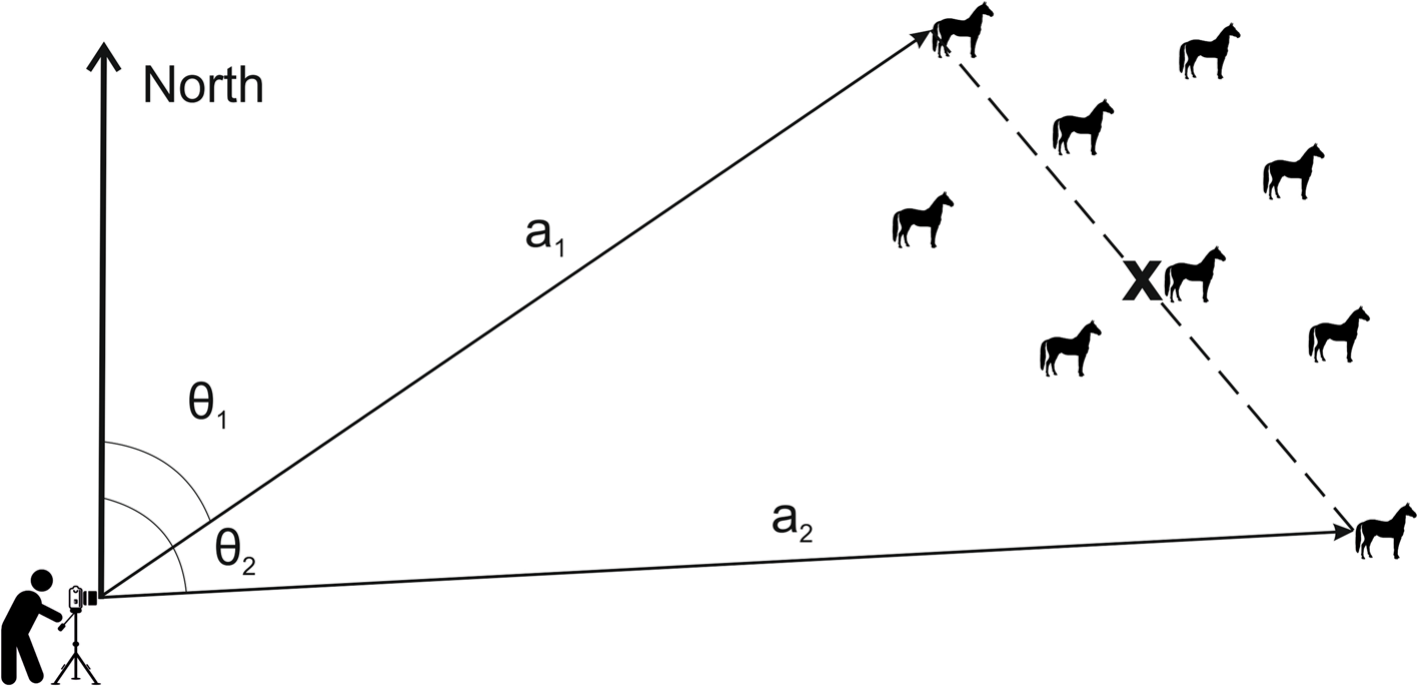
Method followed for the calculation of dispersion and herd location. The two animals which were visually most distant in the group were located by the observer and their distance and angle (between animal and that of true north) from the location of the observer were measured. Dispersion of the group was counted afterwards by basic trigonometric function formulas

The data collection was conducted in three different seasons (late spring, summer, autumn) in the groups described in Table 1. The season was defined according to the consultation with rangers of GGBSPA and local inhabitants (the herders move from winter to summer camps in spring and back in autumn). The studied harems were classified as recently reintroduced (from 2 days up to 3 months after the transport from Europe, fenced area), long-term reintroduced (released after a one-year acclimatization period), and wild-born (born in the wild). The harems were localised by binoculars during daily monitoring routines. Once one of the target harems was localised, the harem was approached and filmed from a close distance (from 150 m to 800 m) by the 4K Panasonic VX1 video camera with tripod. The video recordings were made every day across the whole study period (in Summer, Spring, Autumn respectively) and each observation day was dedicated to one group. We typically spent time from morning till afternoon or from midday to evening with each group. When possible, we aimed to change the group every day and spend equal percentage of morning-afternoon/midday-evening period with each group to collect comparable data in terms of environmental conditions and hours of observation. For the collection of weather conditions Kestrel 4500 Pocket Weather Tracker on a stabilised tripod was used. Weather variables (magnetic heading, true heading, wind speed, crosswind calculation, headwind/tailwind, temperature, wind chill, relative humidity, heat stress index, dewpoint temperature, wet bulb temperature, barometric pressure, altitude, density altitude, cloudiness) were summarised by the device in 10-minute interval together with GPS position of the observation point. The ranges for the data collected in each of the three study periods is shown in Table 2. Distance between the observation point and between each of the two most distant members of the group was measured with a rangefinder Nikon MONARCH 2000 and digital compass, also every 10 minutes so location and dispersion of the group could be calculated later on (Fig. 1). In summer 2018, 45 hours of recording were collected. In late spring 2019, 108 hours of videos were recorded. In autumn 2019, 88.5 hours of videos were recorded. Time of recording was similarly distributed among all studied groups.

**Table 1:** Description of the studied groups

Group	Season/year	Obs. hours	Group experience^**a**^	Individuals	Nr. of breeding ♀	Stability index^**b**^	Age of foals^**c**^
Azaa	Summer/18	18.5	wild-born	1 dom^d^. ♂, 9 ♀, 3♂, 4 foals	6	0.86	46
Mares18	Summer/18	17.5	recently reintroduced	4 ♀	4	0.00	–
Tsetsen	Summer/18	11.2	wild-born	1 dom. ♂, 4 ♀, 3♂	3	0.00	–
Azaa	Spring/19	30	wild-born	1 dom. ♂, 8 ♀, 2♂, 3 foals	5	0.83	26
Hustai1	Spring/19	25.5	long-term reintroduced	1 dom. ♂, 4 ♀	4	0.00	–
Mares19	Spring/19	21	recently reintroduced	3 ♀	3	0.00	–
Tsetsen	Spring/19	31.5	wild-born	1 dom. ♂, 5 ♀, 3♂, 3 foals	4	0.75	28
Azaa	Autumn/19	22.5	wild-born	1 dom. ♂, 6 ♀, 2♂, 5 foals	5	1.00	100
Tanan	Autumn/19	21	long-term reintroduced	1 dom. ♂, 4 ♀	4	0.00	–
Hustai2	Autumn/19	20	recently reintroduced	1 dom. ♂, 3 ♀	3	0.00	–
Tsetsen	Autumn/19	25	wild-born	1 dom. ♂, 5 ♀, 3♂, 3 foals	4	1.00	128

^a^Group experience: recently reintroduced (from 2 days up to 3 months after the transport from Europe, fenced area); long-term reintroduced (after 1 year acclimatization period, released); wild-born (born in the wild). One mare in wild-born Azaa harem was born in captivity in Europe and reintroduced to the GGBSPA in 2004

^b^Counted as the inter-yearly changes in the number of breeding mares (in %; number of mares present in the herd during the previous and the observation year, divided by the number of mares present in one or another year

^c^Average age of foals (in days) at the end of each selected observation

^d^Dominant

**Table 2:** Range of values recorded at each of the study periods for the different weather variables studied (mean is shown in parentheses); and mean time percentage dedicated to each behavioural category studied

	Summer 2018	Spring 2019	Autumn 2019
WS (m/s)	0–8.4 (3.6)	0–12.9 (3.4)	0–8.8 (2.6)
CW (m/s)	0–7.6 (2.8)	0–12.8 (2.6)	0–8.8 (1.8)
HW (m/s)	−5.1 - 6.9 (0.7)	−8.4 - 7.6 (0.0)	−5.9 - 6.7 (− 0.7)
TP (°C)	16.2–32.5 (23.8)	9.0–29.1 (20.2)	3.1–34.6 (18.4)
WC (°C)	15.3–32.5 (23.8)	5.5–29.1 (19.9)	3.1–27.4 (18.0)
RH (%)	1.9–67.3 (25.5)	0.2–69.7 (20.5)	3.0–67.4 (20.8)
HI (°C)	11.0–36.1 (21.6)	7.7–25.7 (17.7)	4.8–31.0 (16.0)
DP (°C)	−24,6–15.1 (− 0.2)	−50.0 - 10.6 (− 6.7)	−23.2 - 8.5 (− 8.3)
WB (°C)	8.1–19.4 (11.4)	2.1–14.3 (8.2)	0.3–13.3 (6.8)
BP (mb)	820.1–837.7 (829.6)	818.0–842.7 (830.9)	816.3–852.7 (836.5)
AL (m)	1583–1746 (1653)	1524–1767 (1640)	1429–1784 (1586)
DA (m)	2051–2774 (2363)	1738–2566 (2217)	1568–2522 (2085)
Clouds (%)	0–75 (15)	0–100 (18)	0–100 (17)
Feeding (%)	15.3	22.0	36.0
Locomotion (%)	14.0	11.7	8.6
Resting (%)	33.6	31.9	29.1
Social (%)	14.1	7.2	7.2
Other (%)	22.9	27.3	19.1

*WS (m/s)* windspeed, *CW (m/s)* crosswind calculation, *HW (m/s)* headwind/tailwind, *TP (°C)* temperature, *WC (°C)* windchill, *RH (%)* relative humidity, *HI (°C)* heat stress index, *DP (°C)* dewpoint temperature, *WB (°C)* wet bulb temperature, *BP (mb)* barometric pressure, *AL (m)* altitude, *DA (m)* density altitude, clouds (location) = % of cloud cover in the place of observation

For categorisation of the behaviours, an ethogram established during the initial data collection was used and complemented with other published material for horses [42, 43]. The ethogram (Table 3) consisted of 5 Categories (feeding, locomotion, resting, social and other) which included specific behaviours. The category Other was computed to calculate accurately the percentage of the previous categories but was not used in further analyses. The average time dedicated to each behavioural category in each studied period is shown in Table 2. No interactions between selected herds or competition for resources was observed along the study.

**Table 3:** Ethogram showing the categories used for describing the behaviour of the studied Prezewalski’s horses

Category	Behaviour	Definition
**Feeding**	Feeding	Food acquisition and investigation, very slow movement connected to feeding is categorized as feeding and not as walking.
**Locomotion**	Lead	Movement to a particular direction while leading other group members, may be accompanied by head movements (up and down) and/or herding.
	Follow	Move along the path of another horse (typically leading mare), usually at the same gait as the horse being followed. There is no attempt to direct the movement, attack, or overtake the leading horse.
	Running	Fast movement to a particular place, fast movement towards and object or other horse is categorized as approach. Fast movement while following or leading some horse(s) is categorized as lead/follow.
	Walking	Slow movement to a particular place, very slow movement connected to feeding is categorized as feeding, slow movement towards and object or other horse is categorized as approach, slow movement while following or leading some horse(s) is categorized as lead/follow.
	Leaving the group	Movement performed in order to increase the distance of an individual from a group resulting in the group abandonment. The abandonment might last from several hours to days or become permanent
	Coming back	Movement performed in order to decrease the distance of an individual from a group resulting in connection to the group. This behaviour typically follows leaving the group.
**Resting**	Standing alert	Rigid stance with the neck elevated and the head oriented toward the object or animal of focus. The ears are held stiffly upright and forward, and the nostrils may be slightly dilated. The whinny vocalization may accompany this stance.
	Stand resting	Standing relaxed with head down.
	Resting	Laying down.
**Social**	Approach	Movement of a horse in attempt to initiate a behaviour towards other horse.
	Play	Behaviour appearing to have no immediate use or function for the animal, involving a sense of pleasure. Various behaviours presented while playing with other individual/s.
	Grooming	Two members standing beside one another, usually head-to shoulder or head-to-tail, grooming (each) other’s neck, mane, rump, or tail by gentle nipping, nuzzling, or rubbing.
	Head resting	Horse is placing it’s head on the other horse’s body.
	Head rubbing	Horse is rubbing it’s head on the other horse’s body. Not connected to sexual activity.
	Olfactory investigation	Olfactory investigation involves sniffing various parts of another horses’ head and/or body. Considered friendly if followed by another friendly behaviour.
	Arched neck threat	Neck tightly flexed with the muzzle drawn toward the chest. Arched neck threats are observed during close aggressive encounters and ritualized interactions.
	Bite threat	No contact is made. The neck is stretched, and ears pinned back as the head swings toward the target horse giving the warning to maintain distance.
	Bite	Opening and rapid closing of the jaws with the teeth grasping the flesh of another horse. The ears are pinned, and lips retracted.
	Chase	One horse pursuing another, usually at a gallop. The chaser typically pins the ears, exposes the teeth, and bites at the pursued horse’s rump and tail. The horse being chased may kick out defensively with both rear legs.
	Ears back	Ears pressed caudally against the head and neck. Typically associated with intense aggressive interaction.
	Fight	Various behaviours associated with fighting, not a single aggressive movement. More than one of the aggressive attempts must be present. Striking, rearing, mounting, lunge, levade, repeated biting/kicking.
	Head bump	In two horses: a rapid lateral toss of the head that forcefully contacts the head and neck of another horse. Usually, the eyes remain closed and the ears forward.
	Herding	Combination of a threat (usually bite) and ears laid back with forward locomotion, apparently directing the movement of another horse.
	Interference	Disruption of combat of horses by moving between the fighting individuals, pushing, attacking, or simple approaching the combatants. One or more horses may simultaneously interfere with an encounter.
	Kick threat	Similar to a kick, but without sufficient extension or force to make contact with the target. The hind leg(s) lifts slightly off the ground and under the body in tense “readiness”.
	Kick	One or both hind legs lift off the ground and extend towards another horse, with apparent intent to make contact.
	Push	Pressing of the head, neck, shoulder, chest, body or rump against another in an apparent attempt to displace target horse.
	Retreat	Movement that maintains or increases an individual’s distance from an approaching horse or a horse initiating some behaviour. The head is usually held low, and ears turned back. The retreat can be at any gait, even very slow and little movement from the initiator.
	Retreat chewing	Moving the lower jaw up and down in a chewing motion. A sucking sound may be made. Typically, the head and neck ate extended, with the ears relaxed and oriented back or laterally.
	Threat	Giving the general appearance of a warning to maintain distance. Threats are typically not directed toward the particular part of the body of other horse.
	Olfactory investigation social	Olfactory investigation involves sniffing various parts of other horses’ head and/or body. Considered agonistic if followed by another agonistic behaviour.
	Copulation	Stallion mounting the female from behind, neck arched over her back and forelegs resting on her sides.
	Copulation attempt	Attempt for copulation without successful completion.
	Defecating over	Defecation over faeces of other group members (typically females) presented by the stallion in a characteristic sequence: sniff faeces, step forward, defecate, pivot or back up, and sniff faeces again.
	Female presentation	Mare presents herself facing away from the stallion, lifting her tail, posturing her body with hind legs slightly apart and often turning her head toward her posterior.
	Head rubbing (sexual)	Stallion proceeds by rubbing his head on the female’s flanks and (or) resting his chin on her back, usually extends his penis out of the prepuce.
	Reproductive tending	Close following of an oestrous female by the male, without the directional driving observed in herding behaviour.
	Sniffing faeces/urine	Sniffing, typically followed by flehmen, defecations and urinations performed by stallion towards faeces of females in oestrus.
	Nursing	Recorded in mares. The foal’s muzzle is in contact with the mare’s udder, movement of milk down the oesophagus is visualized, or the foal is seen to grasp and hold the teat with its lips.
	Suckling	Recorded in foals. The foal’s muzzle is in contact with the mare’s udder, movement of milk down the oesophagus is visualized, or the foal is seen to grasp and hold the teat with its lips.
	Suckling attempt	The foal is trying to reach mare’s udder with its muzzle.
	Vocalization	Emitting a sound produced through the action of respiratory system, used in communication.
**Other**	Defecating	The discharge of faeces from the body.
	Drinking	The action or habit of consuming water.
	Erection	Fully extended and tumescent penis.
	Head bow	Repeated, exaggerated, rhythmic flexing of the neck such that the muzzle is brought toward the point of the breast.
	Insect elimination	Waving of tail, kicking around and/or biting itself or hiding itself under the tail of another horse in attempt of insect elimination.
	Masturbation	Erection with rhythmic drawing of the penis against the abdomen, with or without pelvic thrusting. Solitary or group activity (bachelors).
	Solitary olfactory investigation	Olfactory investigation involves sniffing various parts of a ground or objects.
	Rolling	Dropping from standing to sternal recumbency, then rotating one or more times from sternal to dorsal recumbency. Typically occurs on dusty or sandy areas.
	Urinating	The discharge of urine from the body.

Behavioural Observation Research Interactive Software (BORIS) was used for event logging, video coding and observation of the captured videos. Data from BORIS was transferred to Excel. This data was divided in 10 minutes intervals in order to make them comparable to group position, group dispersion and weather data. Time dedicated to each behavioural category in every 10-minute interval (same as those intervals used for weather and dispersion) was calculated for each individual, then summarised for each group and finally, a percentage representation of each behavioural category was calculated.

### Data analysis

All analyses were conducted in IBM® SPSS® Statistics 27. Pearson’s correlations among all the weather variables were studied (Table S1, Additional file 1), and with the percentage representation of each studied behavioural categories (Table S2, Additional file 1), were conducted in order to measure the statistical relationship between these variables. Due to the high degree of correlation observed among the weather variables, the raw variables were grouped by Principal Component Analysis using a varimax rotation procedure. Six variables with an eigenvalue higher than 1 were selected: F1-T related to temperature, F2-H related to humidity, F3-Mag related to the magnetic heading, F4-Alt related to altitude, F5-Wind related to wind speed, and F6-Cloud related to cloudiness. The contribution of the original variables to the extracted factors, eigenvalue and percentage of variance explained by each new factor is shown in Table 4.

**Table 4:** Scores of the six weather variables used in the factor analysis

	F1-T	F2-H	F3-Mag	F4-Alt	F5-Wind	F6-Cloud
**% Variance explained**	28.234	13.653	13.511	13.414	11.839	11.300
**Eigenvalue**	4.517	2.185	2.162	2.146	1.894	1.808
**MG (mag)**	0.099	0.100	**0.940**	0.099	0.172	0.047
**TR (True)**	0.099	0.100	**0.940**	0.099	0.172	0.047
**WS (m/s)**	0.042	−0.045	0.135	0.069	**0.955**	0.054
**CW (m/s)**	0.039	0.027	0.182	0.094	**0.942**	0.082
**HW (m/s)**	−0.023	0.016	0.493	0.042	0.017	0.081
**TP (°C)**	**0.984**	−0.149	0.028	0.073	0.041	−0.008
**WC (°C)**	**0.987**	−0.132	0.013	0.064	− 0.014	− 0.010
**RH (%)**	− 0.393	**0.866**	0.076	−0.011	− 0.050	0.219
**HI (°C)**	**0.991**	−0.026	0.046	0.079	0.044	0.015
**DP (°C)**	0	**0.962**	0.115	0.090	0.013	0.099
**WB (°C)**	0.740	0.631	0.114	0.086	0.020	0.146
**BP (mb)**	−0.182	−0.056	−0.124	**−0.970**	− 0.086	0.015
**AL (m)**	0.182	0.057	0.124	**0.970**	0.085	−0.013
**DA (m)**	**0.889**	−0.038	0.079	0.444	0.067	0.001
**Clouds (location)**	−0.053	0.143	0.055	−0.034	0.075	**0.931**
**Clouds (whole)**	0.094	0.134	0.160	0.009	0.060	**0.917**

The significant structuring variables (> 0.8, following [44]) are indicated in bold characters

*MG (mag)* magnetic heading, *TR (True)* true heading, *WS (m/s)* windspeed, *CW (m/s)* crosswind calculation, *HW (m/s)* headwind/tailwind, *TP (°C)* temperature, *WC (°C)* windchill, *RH (%)* relative humidity, *HI (°C)* heat stress index, *DP (°C)* dewpoint temperature, *WB (°C)* wet bulb temperature, *BP (mb)* barometric pressure, *AL (m)* altitude, *DA (m)* density altitude, clouds (location) = % of cloud cover in the place of observation, clouds (whole) = % of cloud cover in the observable surrounding

Generalized Mixed Models were built in order to study the influence of the 6 selected weather factors, the group dispersion and the season on the percentage representation of each of the four selected behavioural categories. Data structure was set based on group and period, since most of the studied groups were studied in different periods. Group also entered the model as random factor. Data was also weighed based on the number of adult horses in the herd, which differed between the herds observed. Gamma distribution with log function was set for the models. From all the possible solutions of the model, we selected those with all significant or marginally significant variables and lower AIC value.

One-way ANOVA with post-hoc Tukey test was used to detect differences between the studied groups in the display of the studied behaviours, separately for each studied season.

## Results

**Fig. 2:**
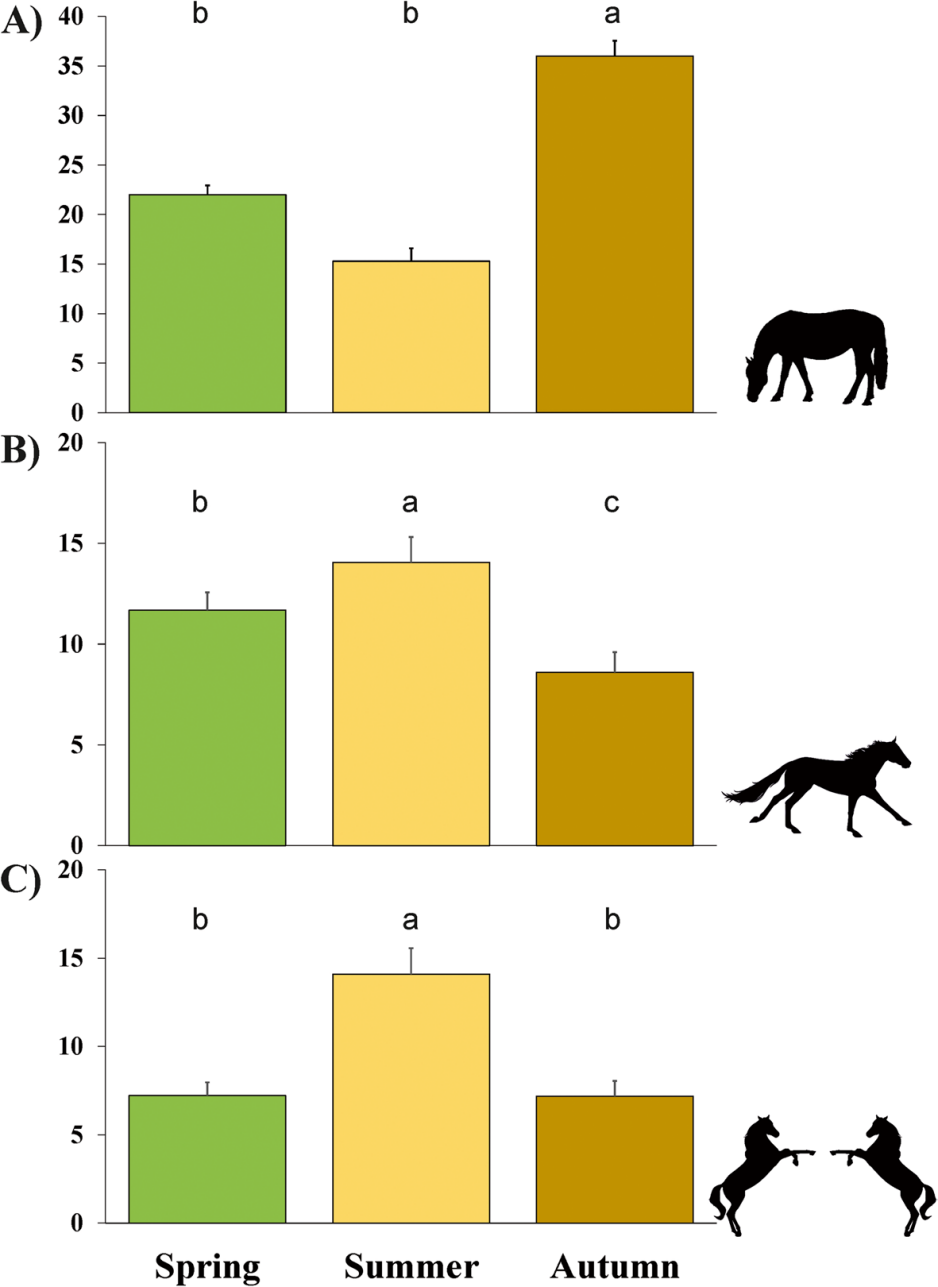
Influence of the season (green = spring, beige = summer, brown = autumn) on the percentage representation of each studied behavioural categories (**A** = feeding, **B** = locomotion, **C** = social). **a, b, c** superscripts indicate significant differences after Tukey test

The models selected for each of the behavioural categories studied is shown in Table 5. The occurrence of feeding behaviour significantly increased with cloudiness (t = 2.013) and under windy conditions (t = 1.987) and was also more frequent in autumn compared to spring and summer (Fig. 2a). Locomotion was positively explained by temperature (t = 3.251) and cloudiness (t = 1.923), and also varied with the period being higher in summer compared to spring and autumn (Fig. 2b). The occurrence of resting behaviour decreased with altitude (t = − 2.892, i.e., the animals preferred to rest in lowlands) and with cloudiness (t = − 3.291), and the dispersion of the group was lower (t = − 2.920, i.e., the animals were closer to each other while resting). Horses showed increased social interactions under higher temperature (t = 2.633). Social interactions were also affected by period, being more frequent in summer compared to spring and autumn (Fig. 2c).

**Table 5:** General linear mixed models (GLMM) assessing the effects of weather variables on the occurrence of different behavioural categories displayed by horses

	Feeding	Locomotion	Resting	Social
**df**	4, 1119	4, 436	3, 1205	4, 405
**F1-T**	ns	F = 10.569 *p* = 0.001	ns	F = 6.932 *p* = 0.009
**F2-H**	ns	ns	ns	ns
**F3-Mag**	ns	ns	ns	ns
**F4-Alt**	ns	ns	F = 8.361 *p* = 0.004	ns
**F5-Wind**	F = 3.950 *p* = 0.047	ns	ns	ns
**F6-Cloud**	F = 4.051 *p* = 0.044	F = 3.697 *p* = 0.055	F = 10.829 *p* = 0.001	ns
**Dispersion**	ns	ns	F = 8.527 *p* = 0.004	ns
**Period**	F = 65.293 *p* < 0.001	F = 17.835 *p* < 0.001	ns	F = 3.367 *p* = 0.026

FX-X = Factors after PCA analysis (see Table 4) related to temperature, humidity, magnetic heading, altitude, wind speed, and cloudiness respectively

*ns* not significant

**Fig. 3:**
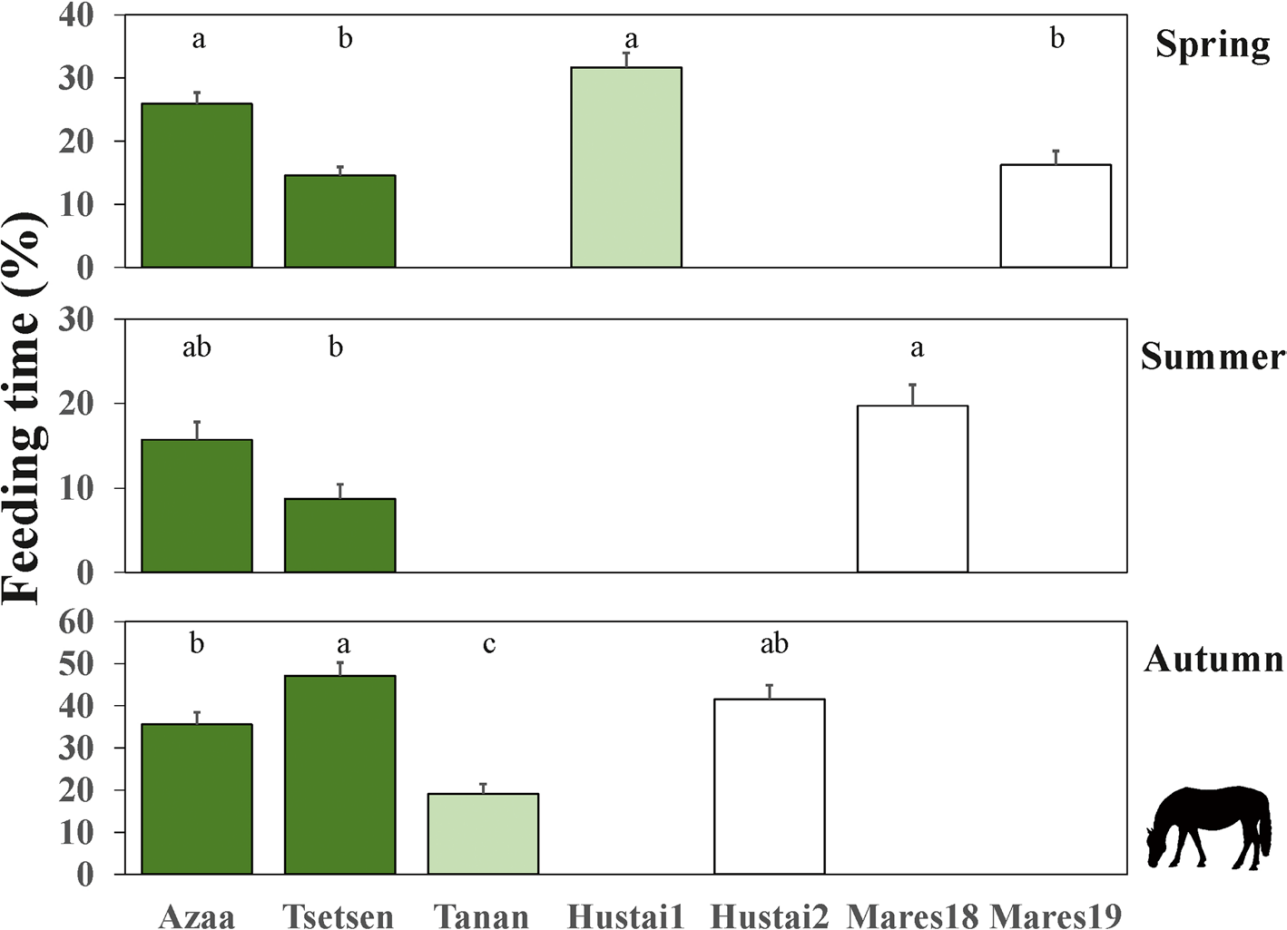
Differences between specific harems for the time spent feeding. The harems are grouped in the figures according to their origin and experience in the area (wild-born harems in dark green; long-term released harems in light green; recently reintroduced harems in white). **a, b, c** superscripts indicate significant differences after Tukey test

**Fig. 4:**
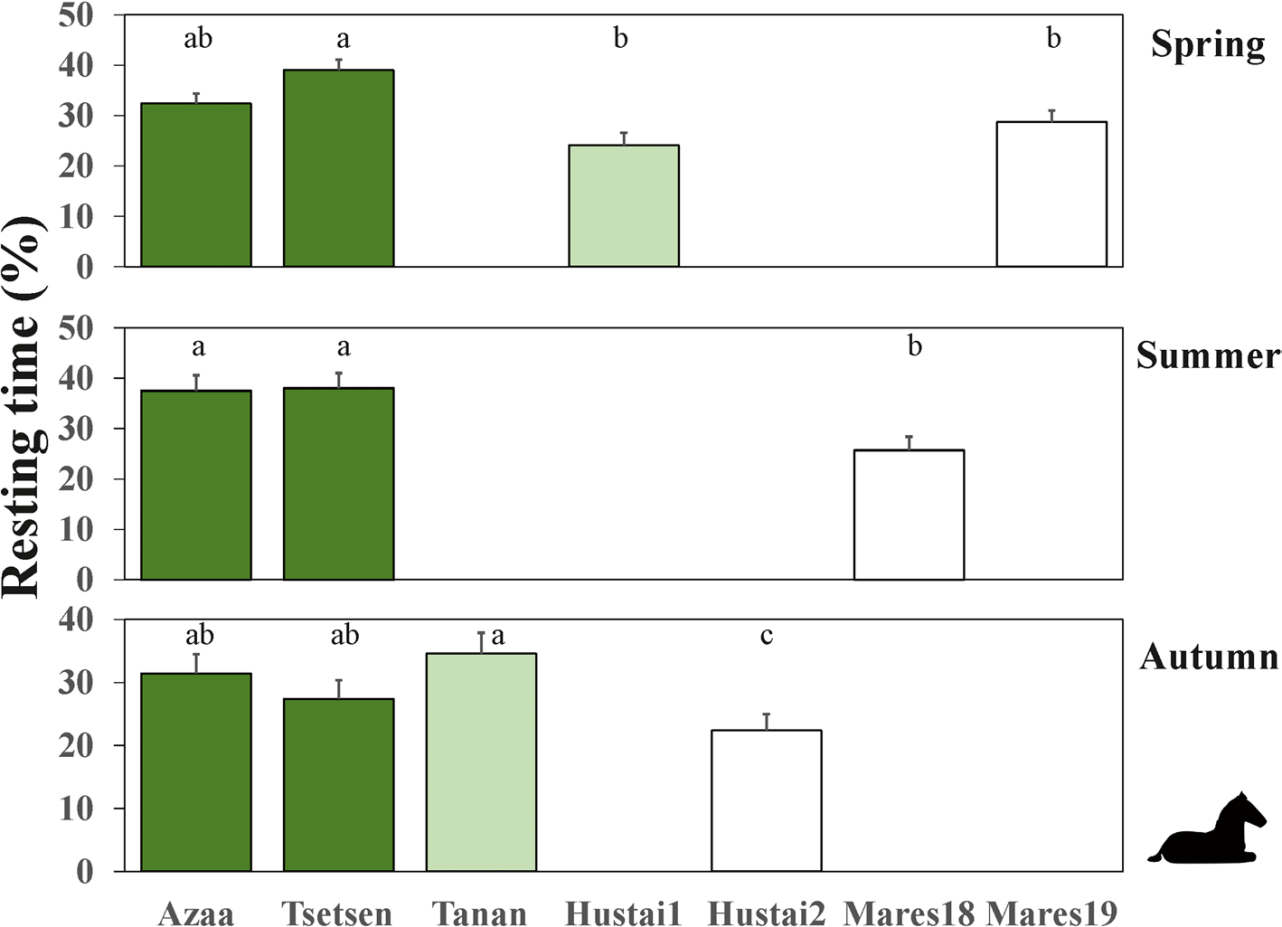
Differences between specific harems for the time spent resting. The harems are grouped in the figures according to their origin and experience in the area (wild-born harems in dark green; long-term released harems in light green; recently reintroduced harems in white). **a, b, c** superscripts indicate significant differences after Tukey test

**Fig. 5:**
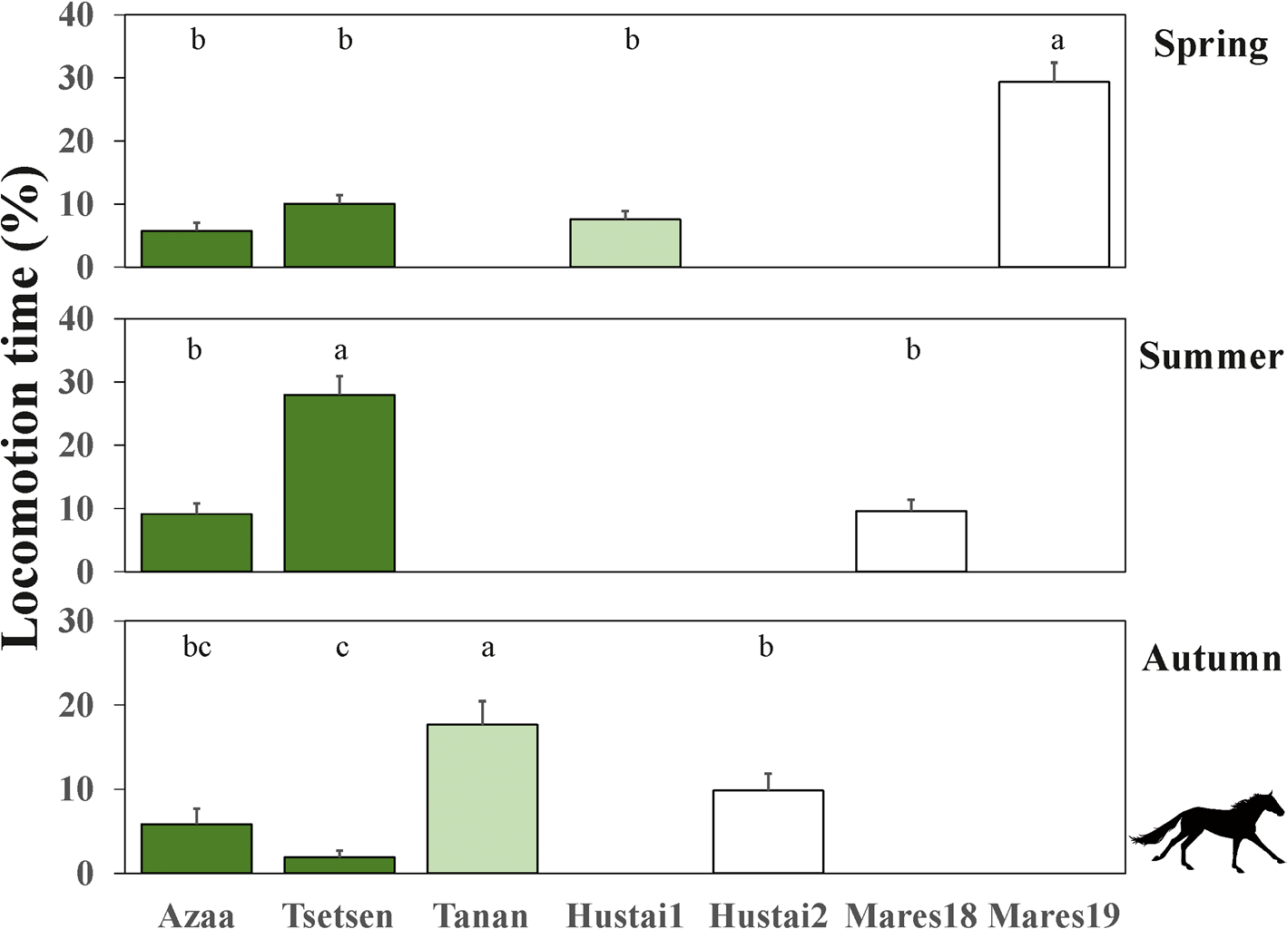
Differences between specific harems for the time spent on locomotion. The harems are grouped in the figures according to their origin and experience in the area (wild-born harems in dark green; long-term released harems in light green; recently reintroduced harems in white). **a, b, c** superscripts indicate significant differences after Tukey test

**Fig. 6:**
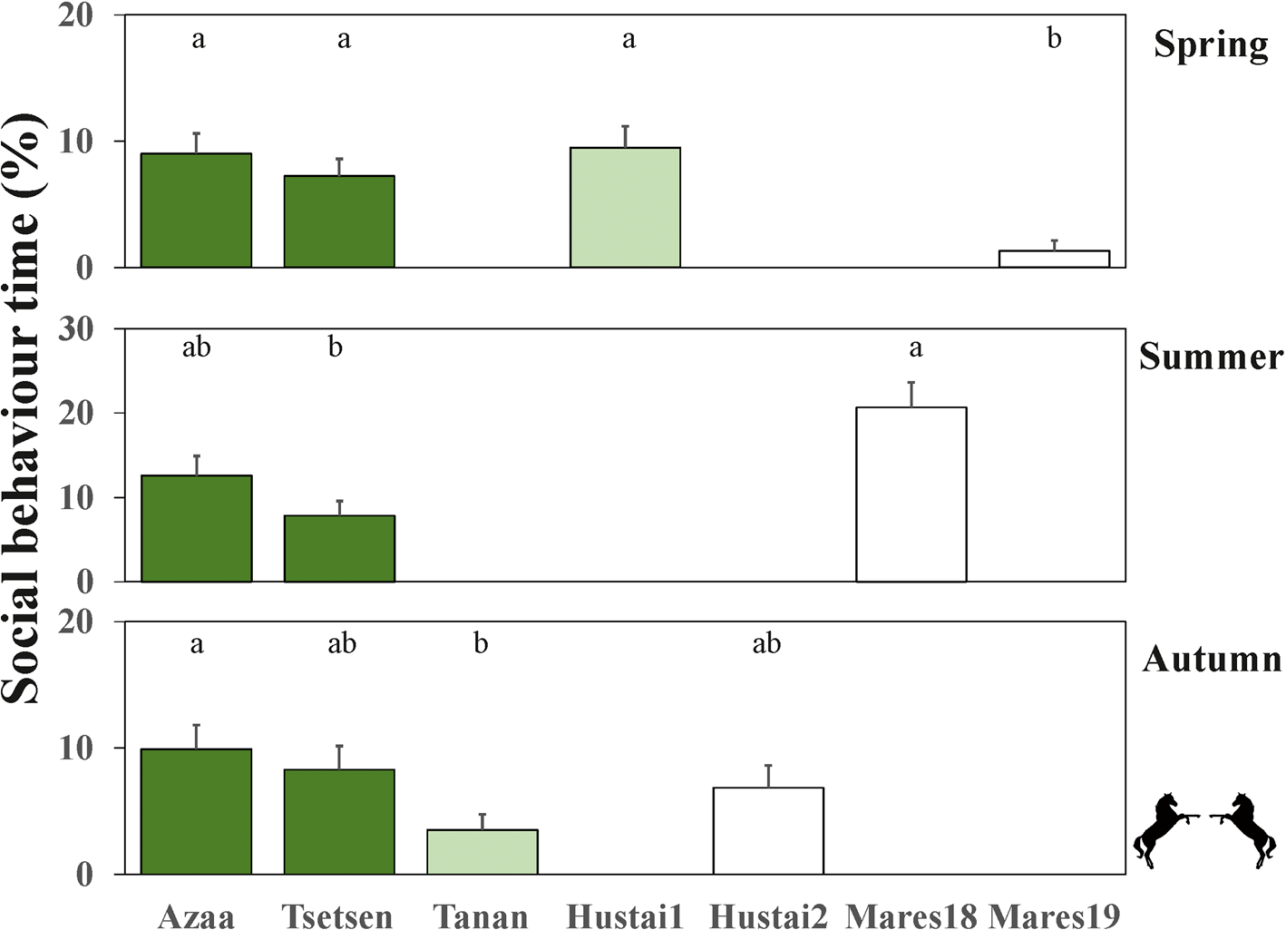
Differences between specific harems for the time spent on social interactions. The harems are grouped in the figures according to their origin and experience in the area (wild-born harems in dark green; long-term released harems in light green; recently reintroduced harems in white). **a, b, c** superscripts indicate significant differences after Tukey test

ANOVA analyses showed differences in the display of the studied behaviours among the studied harems. Overall inter-harem differences in spring were found for feeding (F = 19.613, *p* < 0.001), locomotion (F = 33.060, *p* < 0.001), resting (F = 8.690, *p* < 0.001) and social (F = 4.928, *p* = 0.002). In Summer, inter-harem differences were found for feeding (F = 5.295, *p* = 0.006), locomotion (F = 23.394, *p* < 0.001), resting (F = 5.370, *p* = 0.005), and social (F = 5.957, *p* = 0.003). In Autumn, inter-harem differences were found for feeding (F = 17.405, *p* < 0.001), locomotion (F = 12.269, *p* < 0.001) and resting (F = 2904, *p* = 0.034), but not for social (F = 2.484, *p* = 0.060). Differences between specific harems are shown through superscripts in the figures, respectively for feeding (Fig. 3), locomotion (Fig. 4), resting (Fig. 5) and social (Fig. 6). These differences also show interesting patterns when grouping the harems according to their origin and experience in the area, thus the harems are grouped in the figures according to that (wild-born harems in dark green; long-term released harems in light green; recently reintroduced harems in white).

## Discussion

### Weather effect

Weather conditions are known to affect the behaviour of animals and the response of animals to the environment is vital for their survival. That is especially true for Mongolia’s continental and arid climate, defined by extreme temperature and precipitation variability. Our results indicate that there are seasonal changes in behaviour of Przewalski’s horses, and that weather conditions influence the behaviour of the species. These findings may help for future conservation of endangered Przewalski’s horses, especially considering global climate change.

During our study, occurrence of feeding behaviour significantly increased with cloudiness and during windy conditions and was also more frequent in autumn compared to spring and summer. This result complements the research conducted by Van Dierendonck and Wallis de Vries [25] who described that the maximum number of hours spent grazing in free-living Przewalski horses in Mongolia was documented during fall and winter seasons. Souris et al. [45] described, that feeding is negatively influenced by temperature, this corresponds with our result as in autumn the temperature is typically lower than in summer and late spring. As described by Berger et al. [24] food intake accounts for 40% of total activity of Przewalski’s horses in the summer season (the lowest amount of food intake through the year) which also fits with our results. However, during their research horses spent 62% of time feeding throughout the spring season, representing the highest level of the year. This outcome is contradictory to our result and may be explained by the fact that Berger et al. [24] observed horses in semi-reserve, possibly with more favourable climatic conditions and feed composition. In grasslands of central Asia, the leaf/stem ratio of plant decreases with rising temperatures, promoting the formation of structural carbohydrates, resulting in reduced digestibility of pasture fodder [46]. For this reason, Przewalski’s horses in Gobi might be forced to dedicate more time to grazing in autumn, as the quality of forage after warm summer is considerably lower in these areas.

In the GGBSPA, Przewalski’s horses live on the edge of their original habitat [5, 7, 8] and are highly water limited [36]. The need to stay in relative proximity to water points to be able to drink at least two times per day is forcing them to feed on non-preferred or even normally avoided plants during warmer days. Only after the sunset and during cooler climatic conditions (windy and cloudy weather) they are able to move to more favourable feeding areas [36]. Even during late spring, the temperatures could be considerably high (reaching up to 26 °C, National Statistical Office of Mongolia [41]), limiting the movement of horses. The proximity of mostly undesirable feed sources and inability to move to different areas might cause horses to switch from feeding to alternative behaviours.

Wind plays an important role in habitat use and feeding behaviour of ungulates. During the warm season, ungulates often move to windy and sparsely vegetated sites to avoid biting insects [47]. Activity of biting insects is positively correlated with air temperature and negatively influenced by wind speed [48]. Therefore, windy weather provides horses of Gobi with the opportunity to feed undisturbed and to choose areas preferred for feeding without the necessity of searching shelter from insects. This phenomenon has been already described in previous research stating that serious harassment from biting insects might decrease feed intake in ungulates and resting period, impacting their well-being and body condition [49, 50].

As described in previous research, Przewalski’s horses significantly decrease their metabolic rate during the winter season to cope with food scarcity and harsh weather [51–53]. Therefore, the increase in feeding activity during autumn might be explained by the attempt to make the most of the still available pasture to prepare for upcoming rough climate conditions.

Horses observed in our research showed higher locomotion activity in summer compared to spring and autumn, and their movement was positively explained by temperature and cloudiness. This outcome is consistent with the research by Arnold et al. [54] who stated that Przewalski’s horses in semi-natural conditions present much lower locomotion activity in the cold season in comparison to spring and summer season. However, previous research conducted in the GGBSPA was contradictory to our results and stated that locomotion was more or less constant over the whole observation period and did not change with temperature (May to September, [45]). Nonetheless that study was done considering only recently released horses. Blood-sucking insects are very possibly a significant factor influencing locomotive behaviour of Przewalski’s horses in summer forcing them to frequently move and seek shelter from insect bites. As demonstrated by Blank [47] the largest proportion of insect-repelling behaviour in ungulates occurs during the warm and windless summer season, which is the time of year when most activity of biting insects occurs. Ungulates frequently move to specific landscape features to find high and unvegetated sites with reduced temperatures and higher wind speeds to avoid insect.

The horses preferred to rest in the lowlands during our investigation, and the frequency of resting decreased with cloudiness. While resting, horses maintained lower distance among each other. Research conducted on Przewalski’s horses from May to September [45] and research conducted on domestic horses year-round [55] found that resting is positively influenced by temperature. Resting behaviour usually implies lower metabolic rate, and a reduction in metabolic rate is a typical reaction to extreme temperatures [51]. In Gobi, where almost no shadow exists, cloudy weather may provide relieve from constant radiant heat and allow horses to stop resting and engage in more active behaviours.

Resting behaviour is heavily influenced by feed availability. It lasts longer when the feed sources are abundant [25, 24, 56, 57] and is almost non-existent when there is not enough feed [25, 24, 56–59]. The positive relationship between resting and lowlands, as, in the studied area, food is usually more plentiful and of higher quality in lowlands than at higher altitudes, could be explained by this fact (1550–1805 mamsl, own data). This fits with the research of Heintzelmann-Gröngröft [60] who stated that it is typical for wild horses to rest in open grasslands.

Studies measuring spatial proximity in resting horses showed significant variations in the distance measured between individuals [61–66]. However, in ungulates vulnerable to predation (such as the Przewalski’s horses in Gobi [67]) greater group cohesion supports the dilution effect [68, 69] and increases confusion in predators [70, 71] by creating “safety in numbers”. When group members are closer to each other, the predator gets more confused, and it might be harder to aim the attack on a particular animal [72]. Lower group dispersion observed during resting might be explained as anti-predatory behaviour (higher vigilance) in wild Przewalski’s horses.

### Social interactions

During our research, horses showed increased social interactions under warmer weather conditions. Social interactions were also affected by period, being more frequent in summer compared to spring and autumn. Berger et al. [24] stated that over the period of the year, the general pattern of activity and feeding of semi-wild group of Przewalski’s horse females was closely linked to the time of sunset and sunrise and during daytime most of the social activity occurred.

In some mammalian species, no correlation was found between weather and social interactions [73]. However, in some other [74–76] including ungulates [77, 78] the social interactions might decrease with increasing environmental pressure to reduce the risk of thermal stress. Following this logic, horses in Gobi should engage less in interactions during warm weather conditions and during summer season, when temperatures are generally high, and environment is more demanding than in autumn and spring. However, we observed opposite trend. It could be explained by the fact that during the warm season, the insect harassment is the highest as described by Blank [47] and therefore horses might tend to engage more in social insect repelling behaviour, such as grooming (although we did not distinguish between positive and agonistic interactions in our analyses). The higher frequency of social interactions could not be explained by sexual activity of the selected groups, as it was the highest in spring period during our research (spring: 31 sexual interactions observed; summer: 7; autumn: 22; own data). This result is in contradiction to the study by Stevens [79] who described that the number of social interactions in feral horses was significantly higher in the breeding period.

Przewalski’s horses have been translocated to a variety of environments, most typically to mesic habitats and semi-desert areas [8]. True arid-living species have phenotypic, physiological, and behavioural characteristics to deal with high heat and water stress [80, 81]. Przewalski’s horses, as a mesic evolved species, are unlikely to have structural or physiological characteristics that would help them to survive in the desert. However, behavioural mechanisms like social structure [82], daily budget [83], and locomotion patterns [84] may be modified to allow them to cope with these relatively unfamiliar and severe environments and they can thrive in arid environments as long as they have regular access to water sources [25].

### Group behavioural patterns

We observed an interesting pattern when comparing occurrence of the selected behaviours in different studied groups: The free-ranging, wild-born groups Azaa and Tsetsen shared common behavioural pattern for feeding in summer. However, in spring and autumn, they differed. These differences could be caused by the fact that Tsetsen is an inexperienced harem-holder, and his harem had not existed for a significant period when our observations were conducted. His first harem (Tsetsen) was formed in 2018 only 2 months before the start of our fieldwork. As described by Klimov [85] a harem led by an inexperienced stallion might shift to anomalous behaviours and might not present typical daily activity budget of stable groups.

Moreover, the feeding behaviour displayed by Hustai1 harem, which consisted of mares brought in summer 2018 and a wild stallion Hustai, was very similar to that of the wild-born and stable Azaa harem. Hustai1 harem showed such similar behaviour to that of the Azaa harem after approximately 1 year, supporting the research by Scheibe et al. [38] who found that Przewalski’s horses adapt to novel conditions and start to present the typical yearly behavioural pattern of wild-born horses living in the native habitat 1 year after being translocated.

Nevertheless, when the same females were led by Tanan stallion (who defeated Hustai stallion) the time dedicated to feeding greatly differed from both wild-born groups. Even if these mares were in Gobi for more than 1 year and clearly started to present the typical behavioural pattern of wild-born horses in spring, their behaviour was indeed influenced by the change of the stallion. Stallions may play an important role when exploring a novel environment [86] and have the ability to play a unique role in the decision-making process. Indisputably, holding females together is part of their reproductive strategy, and specialised acts such as herding allows them to affect the behaviour of the entire group [87]. The fact that Hustai2 harem, consisting of the stallion Hustai and mares brought in spring 2019, shared similar feeding pattern with both wild-born groups in autumn, support the stallion influence interpretation.

The wild-born, free-ranging groups Azaa and Tsetsen shared common behavioural pattern for resting during all studied periods. Both, Azaa and Tsetsen, shared similar resting pattern with free-ranging Tanan in autumn, and in spring, Azaa shared the pattern with free-ranging Hustai1 and Mares19 (enclosure). Mares18 (enclosure) differed from both, Azaa and Tsetsen and Hustai2 (enclosure) different from all three free-ranging groups in autumn. This variation in resting time might be caused by the fact, that Hustai2 and Mares18 were observed only in the enclosure. As described by Keiper and Receveur [88], size of the area used by horses has a significant influence on time dedicated to resting and in general, horses in smaller enclosures tend to rest less. Nonetheless, one of the enclosed groups, Mares19, did not differ from free-ranging Azaa and Hustai1 in the resting pattern in spring.

Keiper and Receveur [88] also found that locomotion in Przewalski’s horses is influenced by the size of enclosure. However, we only observed this trend in spring, when Mares19 (enclosure) differed in the locomotion pattern from free-ranging Azaa, Tsetsen and Hustai, who shared similar pattern. During our research in summer and autumn this trend was not observed.

In spring, we found that free-ranging groups of Azaa, Tsetsen and Hustai1 shared similar pattern in social behaviour, and enclosed Mares19 differed from these three groups. In previous research it was described that area size influences the rate of social interactions among horses, as individuals in smaller enclosures were reported to engage more in social interactions than those on large pastures [88, 89]. Nonetheless, we did not find such differences between enclosed and free-ranging groups in autumn and summer.

During our research, we detected influence of the harem characteristic on the activity budget. One of the factors was harem origin and experience. It was described in previous research that after the release, wild horses might show differences from the typical pattern in specific behaviours, such as locomotion and resting up to 1 year [38], or even 2 years after the release [90]. In general, differences from the typical annual behavioural pattern of Przewalski’s horses presented in our results can be explained by the period of adaptation of recently reintroduced horses to the seasonal change of climatic and nutritional conditions, as also described by Scheibe et al. [38], and Boyd and Bandi [90].

Nonetheless, we also detected influence caused by the characteristics of harem holder (dominant stallion) and area size on the behavioural pattern of Przewalski’s horses.

During our research, only two groups (Azaa and Tsetsen) were observed in all three seasons as the structure of other groups was changing through the observation period or included the observation of horses transported from Europe in 2019 (i.e., new groups). Winter monitoring was not possible due to low accessibility to the area (frozen paths) and impossibility to record the herds due to the extremely low temperatures. For this reason, we emphasize a need for further research concerning more groups (if possible, same ones) and all seasons (including winter).

### Implications for conservation

It was stressed by several studies that appropriate response of animals to the environment is vital for their survival [42, 91, 92].

The disastrous winter of 2009 and 2010, resulting in the loss of the 60% of Przewalski’s horse population, demonstrated how sensitive are the small and spatially restricted populations to severe climatic and environmental changes [27]. As stated by Slotta-Bachmayr [93] the severity level of natural climatic conditions has the highest influence on extinction risk and population size of Przewalski’s horses in the Great Gobi B, according to population simulation model VORTEX. The extinction risk for severe weather disasters measured by Slotta-Bachmayr [93] was 37%, even for initial population size larger than 500 individuals. The Przewalski’s horse population in the GGBSPA is still significantly smaller and thus highly susceptible to severe weather conditions.

According to the 2014 Mongolia Second Assessment Report on Climate Change, the impact of climate change is already visible in Mongolia and this country is highly vulnerable being ranked 8th among over 100 countries according to the Global Climate Risk Index. Winters are becoming warmer and snowier; the temperature is expected to increase continuously in all seasons and there is a high probability of climate anomalies happening more often in future [94]. In this study we found out that there is an important link between the behaviour of Przewalski’s horse and different weather factors. These findings might assist in the successful selection of future reintroduction sites and in further conservation of the species, especially in the view of intensifying climate change and alteration of weather patterns.

## Supplementary Information

**Additional file 1: Table S1.** Pearson’s correlations among all weather variables measured during the research. **Table S2.** Pearson’s correlations among all the weather variables and the percentage representation of each studied behavioural categories.

## Data Availability

The datasets used and/or analysed during the current study are available from the corresponding author on reasonable request.

## Abbreviation

GGBSPA: Great Gobi B Strictly Protected Area
