# Supplementary material for: Influence of weather on the behaviour of reintroduced Przewalski’s horses in the Great Gobi B Strictly Protected Area (Mongolia): implications for conservation
Source: BMC Zool. 2022 Jun 9;7:32. doi: 10.1186/s40850-022-00130-z (PMC10127430; doi:10.1186/s40850-022-00130-z)
Supplement: Supplementary file 1 — Additional file 1: Table S1. Pearson’s correlations among all weather variables measured during the research. Table S2. Pearson’s correlations among all the weather variables and the percentage representation of each studied behavioural categories. [file 40850_2022_130_MOESM1_ESM.docx]

**Influence of weather on the behaviour of reintroduced Przewalski´s Horses in the Great Gobi B Strictly Protected Area (Mongolia). Implications for conservation.**

Anna BERNÁTKOVÁ^1^, Ganbaatar OYUNSAIKHAN^2^, Jaroslav ŠIMEK^3^, Martina KOMÁRKOVÁ^1^, Miroslav BOBEK^3^, Francisco CEACERO^1,*^

^1^ Faculty of Tropical AgriSciences, Czech University of Life Sciences Prague, Czech Republic.

^2^ Great Gobi B Strictly Protected Area, Takhin Tal, Mongolia.

^3^ Zoo Praha, U Trojského zámku 120/3, Prague, Czech Republic.

* **Correspondence:** ceacero@ftz.czu.cz

**Supplementary Material**

**Table S1.** Pearson´s correlations among all weather variables measured during the research.

|  | TR (True) | WS (m/s) | CW (m/s) | HW (m/s) | TP (°C) | WC (°C) | RH (%) | HI (°C) | DP (°C) | WB (°C) | BP (mb) | AL (m) | DA (m) | Clouds (location) | Clouds (whole) |
| --- | --- | --- | --- | --- | --- | --- | --- | --- | --- | --- | --- | --- | --- | --- | --- |
| Dispersion | 0.002 | 0.027 | 0.027 | 0.049* | -0.120*** | -0.119*** | -0.041 | -0.132*** | -0.103*** | -0.179*** | 0.310*** | -0.309*** | -0.228*** | -0.015 | -0.042 |
| TR (True) |  | 0.294*** | 0.341*** | 0.271*** | 0.120*** | 0.099*** | 0.120** | 0.151*** | 0.214*** | 0.255*** | -0.251*** | 0.252*** | 0.212*** | 0.130*** | 0.226*** |
| WS (m/s) |  |  | 0.878*** | 0.112*** | 0.095*** | 0.039 | -0.083** | 0.097*** | -0.001 | 0.054* | -0.170*** | 0.170*** | 0.145*** | 0.114*** | 0.130*** |
| CW (m/s) |  |  |  | 0.154*** | 0.085** | 0.031 | -0.006 | 0.098*** | 0.072** | 0.106*** | -0.201*** | 0.201*** | 0.153*** | 0.151*** | 0.167*** |
| HW (m/s) |  |  |  |  | 0.001 | -0.012 | 0.077** | 0.020 | 0.108*** | 0.092*** | -0.095*** | 0.095*** | 0.046 | 0.065** | 0.138*** |
| TP (°C) |  |  |  |  |  | 0.995*** | -0.516** | 0.989*** | -0.134*** | 0.642*** | -0.249*** | 0.249*** | 0.919*** | -0.076** | 0.068** |
| WC (°C) |  |  |  |  |  |  | -0.501** | .984*** | -0.121*** | 0.652*** | -0.235*** | 0.234*** | 0.910*** | -0.081** | 0.065** |
| RH (%) |  |  |  |  |  |  |  | -0.402*** | 0.824*** | 0.301*** | 0.031 | -0.030 | -0.382*** | 0.347*** | 0.284*** |
| HI (°C) |  |  |  |  |  |  |  |  | -0.013 | 0.730*** | -0.265*** | 0.265*** | 0.924*** | -0.034 | 0.106*** |
| DP (°C) |  |  |  |  |  |  |  |  |  | 0.632** | -0.155** | 0.155** | 0.012 | 0.236** | 0.243** |
| WB (°C) |  |  |  |  |  |  |  |  |  |  | -0.267** | 0.268** | 0.683** | 0.186** | 0.311** |
| BP (mb) |  |  |  |  |  |  |  |  |  |  |  | -1.000** | -0.606** | 0.034 | -0.044 |
| AL (m) |  |  |  |  |  |  |  |  |  |  |  |  | 0.606** | -0.033 | 0.046 |
| DA (m) |  |  |  |  |  |  |  |  |  |  |  |  |  | -0.056* | 0.096** |
| Clouds (location) |  |  |  |  |  |  |  |  |  |  |  |  |  |  | 0.782** |

Significance is indicated as *, ** and *** for 0.05, 0.01 and 0.001 levels, respectively.

MG (mag) = magnetic heading; TR (True) = true heading; WS (m/s) = windspeed; CW (m/s) = crosswind calculation; HW (m/s) = headwind/tailwind; TP (°C) = temperature; WC (°C) = windchill; RH (%) = relative humidity; HI (°C) = heat stress index; DP (°C) = dewpoint temperature; WB (°C) = wet bulb temperature; BP (mb) = barometric pressure; AL (m) = altitude; DA (m) = density altitude; clouds (location) = % of cloud cover in the place of observation; clouds (whole) = % of cloud cover in the observable surrounding.

**Table S2.** Pearson´s correlations among all the weather variables and the percentage representation of each studied behavioural categories.

|  | Feeding | Locomotion | Other | Resting | Social |
| --- | --- | --- | --- | --- | --- |
| Dispersion | 0.190*** | -0.055* | -0.065** | -0.058* | -0.049 |
| TR (True) | -0.045 | 0.051* | -0.109*** | 0.076** | 0.018 |
| WS (m/s) | -0.053* | 0.014 | -0.087** | 0.087*** | 0.028 |
| CW (m/s) | -0.003 | 0.010 | -0.106*** | 0.075** | 0.001 |
| HW (m/s) | -0.016 | 0.009 | -0.024 | 0.038 | -0.019 |
| TP (°C) | -0.182*** | 0.108*** | 0.007 | 0.023 | 0.099*** |
| WC (°C) | -0.180*** | 0.103*** | 0.024 | 0.016 | 0.092*** |
| RH (%) | 0.078** | 0.008 | -0.023 | -0.038 | -0.037 |
| HI (°C) | -0.181*** | 0.119*** | 0.001 | 0.019 | 0.097*** |
| DP (°C) | -0.001 | 0.061* | 0.014 | -0.046 | -0.013 |
| WB (°C) | -0.152** | 0.130** | 0.017 | -0.005 | 0.063* |
| BP (mb) | 0.130** | -0.054* | -0.074** | 0.003 | -0.045 |
| AL (m) | -0.129** | 0.054* | 0.074** | -0.003 | 0.045 |
| DA (m) | -0.205** | 0.115** | 0.037 | 0.016 | 0.099** |
| clouds place | 0.094** | -0.014 | -0.071** | -0.042 | 0.027 |
| clouds whole | 0.028 | 0.007 | -0.061* | -0.016 | 0.047 |

Significance is indicated as *, ** and *** for 0.05, 0.01 and 0.001 levels, respectively.

MG (mag) = magnetic heading; TR (True) = true heading; WS (m/s) = windspeed; CW (m/s) = crosswind calculation; HW (m/s) = headwind/tailwind; TP (°C) = temperature; WC (°C) = windchill; RH (%) = relative humidity; HI (°C) = heat stress index; DP (°C) = dewpoint temperature; WB (°C) = wet bulb temperature; BP (mb) = barometric pressure; AL (m) = altitude; DA (m) = density altitude; clouds (location) = % of cloud cover in the place of observation; clouds (whole) = % of cloud cover in the observable surrounding.
